# Gut-Derived Endotoxin and Telomere Length Attrition in Adults with and without Type 2 Diabetes

**DOI:** 10.3390/biom11111693

**Published:** 2021-11-14

**Authors:** Nasser M. Al-Daghri, Saba Abdi, Shaun Sabico, Abdullah M. Alnaami, Kaiser A. Wani, Mohammed G. A. Ansari, Malak Nawaz Khan Khattak, Nasiruddin Khan, Gyanendra Tripathi, George P. Chrousos, Philip G. McTernan

**Affiliations:** 1 Chair for Biomarkers of Chronic Diseases, Biochemistry Department, College of Science, King Saud University, Riyadh 11451, Saudi Arabia; sabdi@ksu.edu.sa (S.A.); ssabico@ksu.edu.sa (S.S.); aalnaami@ksu.edu.sa (A.M.A.); kwani@ksu.edu.sa (K.A.W.); mansari@ksu.edu.sa (M.G.A.A.); mkhattak@ksu.edu.sa (M.N.K.K.); 2 Department of Food Science and Human Nutrition, College of Applied and Health Sciences, A’Sharqiyah University, Ibra 400, Oman; knasiruddin@asu.edu.om; 3 Human Sciences Research Centre, School of Human Sciences, University of Derby, Derby DE22 1GB, UK; g.tripathi@derby.ac.uk; 4 University Research Institute of Maternal and Child Health and Precision Medicine, UNESCO Chair on Adolescent Health Care, National and Kapodistrian University of Athens, 11527 Athens, Greece; chrousos@gmail.com; 5 Department of Biosciences, School of Science and Technology, Nottingham Trent University, Nottingham NG1 8NS, UK

**Keywords:** telomere length, endotoxin, inflammation, type 2 diabetes mellitus

## Abstract

Premature aging, as denoted by a reduced telomere length (TL), has been observed in several chronic inflammatory diseases, such as obesity and type 2 diabetes mellitus (T2DM). However, no study to date has addressed the potential inflammatory influence of the gut-derived Gram-negative bacterial fragments lipopolysaccharide, also referred to as endotoxin, and its influence on TL in low-grade inflammatory states such as type 2 diabetes mellitus (T2DM). The current study therefore investigated the influence of endotoxin and inflammatory factors on telomere length (TL) in adults with (T2DM: *n* = 387) and without (non-diabetic (ND) controls: *n* = 417) obesity and T2DM. Anthropometric characteristics were taken, and fasted blood samples were used to measure biomarkers, TL, and endotoxin. The findings from this study highlighted across all participants that circulating endotoxin (r = −0.17, *p* = 0.01) was inversely associated with TL, noting that endotoxin and triglycerides predicted 18% of the variance perceived in TL (*p* < 0.001). Further stratification of the participants according to T2DM status and sex highlighted that endotoxin significantly predicted 19% of the variance denoted in TL among male T2DM participants (*p* = 0.007), where TL was notably influenced. The influence on TL was not observed to be impacted by anti-T2DM medications, statins, or anti-hypertensive therapies. Taken together, these results show that TL attrition was inversely associated with circulating endotoxin levels independent of the presence of T2DM and other cardiometabolic factors, suggesting that low-grade chronic inflammation may trigger premature biological aging. The findings further highlight the clinical relevance of mitigating the levels of circulating endotoxin (e.g., manipulation of gut microbiome) not only for the prevention of chronic diseases but also to promote healthy aging.

## 1. Introduction

The prevalence of type 2 diabetes mellitus (T2DM) has increased dramatically in recent years, with 1 in 11 adults now estimated to have diabetes, 90% of which is associated with T2DM. This increase has been even more rapid in Asia and the Middle East due to the rise in obesity levels in these populations [1,2].

Beyond the clear risk the weight gain poses, the risk of T2DM is also impacted by ethnicity and genetic and nutritional factors [2,3]. In Saudi Arabia currently, a quarter of adults are diabetic (mostly T2DM), and the prevalence of the disease is predicted to more than double within the next decade [4,5]. Obesity is considered to be a key risk factor in T2DM pathology, partly as adipose tissue expansion leads to changes in how the tissue regulates metabolism and responds to systemic factors, such as nutrients and inflammatory insults [6]. In conditions such as obesity, adipose tissue can mediate an exacerbated inflammatory response, which promotes insulin resistance and pathogenesis of T2DM [7,8]. Furthermore, studies have provided strong evidence that a T2DM metabolic state also leads to decreased telomere length (TL) as monitored through leukocyte telomere length (LTL) analysis, and as such premature aging [9,10,11,12,13]. Yet although obesity and inflammation play pivotal roles in increasing susceptibility to T2DM, the specific mediators contributing to this pathology and associated premature aging have been less apparent.

However, studies assessing obesity, inflammation, T2DM, and other chronic diseases have implicated gut-derived Gram-negative bacterial fragments, referred to as endotoxin, as an inflammatory insult able to enter the circulation and mediate an inflammatory response from adipose tissue [6,14,15]. In fact, previous studies have reported that the concentration of endotoxin in individuals can predict future diabetes [16]. As endotoxin can be used to predict future T2DM risk, noting a diabetic state leads to endotoxin-induced inflammation, endotoxin could represent an important factor associated with premature aging. Studies have shown that manipulation of intestinal flora by either dietary interventions or weight-loss surgery can reduce endotoxin-induced inflammation and give beneficial health outcomes, which may also in the long term influence telomere length shortening [13,17,18,19,20].

Telomeres are a DNA protein complex present at both ends of linear eukaryotic chromosomes. A tandem repeat of TTAGGG present in the telomere plays an important role in protecting genes from nucleolytic degradation, preventing end-to-end chromosomal fusion and maintaining chromosome stability. Telomere length is regulated by telomerase, which adds telomeric repeats to chromosomal ends. Each successive mitotic cell division results in shortened TL, and cellular replication continues until a critical threshold of telomere shortening is reached. Once a somatic cell reaches this threshold point, it becomes senescent or dies [21]. Thus, reduced TL has been hypothesized as a biomarker for aging and age-related disorders [22,23,24]. Studies on TL in the context of metabolic disease have led to the understanding that aberrant systemic lipid profiles and inflammation are correlated with the telomere shortening noted in South Asian subjects with T2DM [12]. A similar influence was also observed in an Arabian population, where significant inverse associations between LTL and metabolic biomarkers of obesity and insulin resistance were observed [25]. These previous studies shed light on the possible role of inflammation and its effect on TL as a marker for predisposition to age-related diseases. A potential underlying mechanism suggested for the association between obesity and shortened telomere length is the occurrence of chronic low-level systemic inflammation. As such, in obesity adipose tissue (AT) increases the production of pro-inflammatory cytokines, and the resulting inflammation enhances leukocyte proliferation rate, which in turn increases telomere attrition regardless of chronological age [26,27]. An increased concentration of circulating endotoxin may initiate the inflammation in obesity and metabolic disorders [28]. However, no study to date has examined the in vivo relationship between endotoxin and telomere length in obese T2DM patients. Therefore, in this study we hypothesized that the level of gut-derived endotoxin, which is increased in patients with T2DM, can promote telomere loss, thus representing a key factor influencing cellular aging. Hence, the aim of this study was to evaluate TL in leukocytes derived from Saudi adults with and without diabetes and to examine the influence of endotoxin and elevated pro-inflammatory markers on TL in T2DM subjects.

## 2. Materials and Methods

### 2.1. Participants

In all, 804 Saudi adults were recruited for this study. The participants were classified into two groups: T2DM group (387 T2DM patients (202 men and 157 women)) and a case control group (417 non-diabetic participants (183 men and 234 women)). The participants with fasting plasma glucose of ≥7 mmol/L were classified as T2DM patients, and those with fasting plasma glucose of ≤6.1 mmol/L were classified as controls. All the participants were recruited from primary care centers throughout Riyadh and the Biomarkers Research Program (BRP), College of Science, King Saud University, Riyadh, Saudi Arabia, as part of a larger database (RIYADH COHORT) as previously described [29,30,31]. Demographic data were collected from all participants using a generalized, pre-structured questionnaire detailing present and past medical history. All participants underwent physical examination and submitted written informed consent before inclusion. Patients taking multivitamins, calcium, cortisone, or other steroids; products with mineral oil, regular antacids, diuretics, phenytoin, and phenobarbital medications; or weight loss drugs were excluded from the study. In addition, patients with gallbladder or gastrointestinal disorders, those with liver problems, and those with evidence of metabolic disease (Paget’s disease or osteomalacia), renal stone disease, hyperparathyroidism, or abnormal levels of calcium, alkaline phosphatase, or phosphorous were excluded. All methods of sampling and protocols were approved by the Ethics Committee and Institutional Review Board (IRB) of the College of Medicine, King Saud University in Riyadh, Saudi Arabia (IRB Ethics case no.8-25-454239). All methods were performed in accordance with the relevant guidelines of the Declaration of Helsinki.

### 2.2. Anthropometry and Blood Collection

Blood samples were taken as required. Anthropometric data and blood pressure were assessed with all other tests as a basis to examine the metabolic risk and cardiovascular risk profile. The anthropometry including weight (kg), height, and hip waist size (cm) was measured using a Digital Pearson Scale (ADAM equipment Inc., Oxford, CT, USA). Systolic and diastolic blood pressure measurements were taken after 15-min rest using a standard mercurial sphygmomanometer. Hypertension was defined based on the criteria used at the time of data collection (≥140/90 mmHg) as well as the use of anti-hypertensive medications [29,31]. Fasting blood samples were collected and harvested for PMBC, serum, and plasma and stored appropriately.

### 2.3. Biochemical Analyses

The fasting plasma glucose (FPG) and lipid profile was measured using a chemical analyzer (Konelab, Vantaa, Finland). LDL/HDL ratio was calculated. Serum leptin, insulin, and tumor necrosis factor (TNF)-α were quantified using Milliplex Map (Millipore, Billerica, MA, USA) in FlexMAP 3D (Luminex Corp, Austin, TX, USA). Minimum detectable concentrations (MDC) were as follows: TNF-α 0.14 pg/mL; insulin 50.9 pg/mL; leptin 85.4 pg/mL. Resistin, angiotensin II (ANG II), adiponectin, and plasminogen activator inhibitor 1 (PAI-1) were analyzed using a separate multiplex kit (Millipore, Billerica, MA, USA). The MDC were as follows: adiponectin 145.4 pg/mL; ANGII 13 pg/mL; PAI-1 1.3 pg/mL; resistin 6.7 pg/mL. The intra-assay variation was 1.4–7.9% and inter-assay variation <21%. Serum C-reactive protein (CRP) was measured using assays (intra-assay precision (4.4–8.3) and inter-assay precision (6.0–7.0)) (R&D Systems, Minneapolis, MN, USA). The homeostasis model assessment of insulin resistance (HOMA-IR) was calculated as fasting insulin (µU/mL) X FPG ((mmol/L)/22.5) [32]. Serum endotoxin was analyzed using commercially available QCL-1000 LAL endpoint assays (Lonza, Morristown, NJ, USA).

### 2.4. Telomere Length (TL) Measurements

TL was examined by quantitative real-time PCR using the IQ cycler (Bio-Rad Laboratories, Hercules, CA, USA) [25,33]. This technique used telomere-specific primers and single-copy control gene primers to compare the number of cycles needed to amplify a product to a set fluorescence threshold [33]. This measurement is important for determining the dynamic and functional importance of length changes in specific telomeres.

### 2.5. Statistical Analysis

Data were analyzed using SPSS (version 22, Chicago, IL, USA). Continuous data were presented as mean ± standard deviation (SD) for normal variables, and non-Gaussian variables were presented in median (1st and 3rd) percentiles. Categorical data were presented as frequencies and percentages (%). All continuous variables were checked for normality using Kolmogorov–Smirnov test. Non-Gaussian variables were log-transformed prior to parametric analysis. Independent *t*-test and Mann–Whitney U were used to compare mean and median differences in Gaussian and non-Gaussian variables. Correlations between variables were done using Pearson’s and Spearman correlation analysis. Analysis of variance was undertaken to determine differences in TL and endotoxin in conjunction with medications consumed by subjects. Stepwise linear regression analysis was undertaken to determine significant predictors of TL and endotoxin in all participants and after stratification according to T2DM status and sex, with age, BMI, WHR, glucose, triglycerides, adiponectin, resistin, leptin, PAI-1, TNF-α, insulin, 25(OH)D, ANGII, CRP, TL, and endotoxin entered as independent variables. *P*-value < 0.05 was considered statistically significant.

## 3. Results

### 3.1. Clinical Characteristics of Participants

Several pre-existing conditions were noted in participants regardless of T2DM status (Table 1). More than half of all participants were obese in both groups, which was undertaken to match groups and was not significantly different between the two groups even considering stratification according to sex. Prevalence of hypertension was significantly higher in the T2DM group (*p* < 0.001) overall as anticipated; this significant difference was driven mainly by the T2DM women cohort (*p* = 0.002). Insulin analogs were the most common medications used by the T2DM cohort (10.6%) followed by metformin (2.9%) and statins (2.1%). The use of anti-hypertensives and use of aspirin were present in both groups but were significantly more common in the T2DM cohort (both *p*-values at <0.001).

The T2DM participants were noted to be older than their controls (*p* < 0.001). The T2DM cohort also had significantly higher WHR, systolic BP, diastolic BP, and circulating triglycerides as compared with the non-diabetic (ND) control cohort (*p*-values < 0.001, < 0.001, 0.001, and 0.002, respectively). When stratified according to sex, male T2DM participants were slightly older with significantly higher WHR, systolic BP, HDL cholesterol, and LDL cholesterol than their ND control counterparts (*p*-values < 0.001, < 0.001, < 0.001, 0.004, and 0.02, respectively). In comparison to their ND controls, the women T2DM participants had significantly higher systolic BP, diastolic BP, and triglycerides (*p*-values < 0.001, 0.007, and < 0.001, respectively) (Table 2).

### 3.2. Telomeric Length, Glycemic Profile, and Pro-Inflammatory Markers

Unadjusted comparisons of clinical markers, including TL, according to T2DM status, as well as between men and women are described in Table 3. In all participants, mean TL was significantly shorter in the T2DM group than ND controls (*p* < 0.001). Consequently, circulating levels of fasting glucose, HOMA-IR, and PAI-1 were also significantly higher in the T2DM group than ND controls (all *p*-values < 0.001). The control ND group in contrast had higher endotoxin, ANG-II, and TNF-α (*p*-values 0.03, < 0.001, and < 0.001, respectively). The rest of the measured parameters were not significantly different from one another (Table 3).

Sub-analysis according to gender revealed that in men and women, levels of fasting glucose, insulin, resistin, ANF II, and CRP were significantly higher in the T2DM group than the ND controls. In men only, mean TL in the T2DM group was significantly shorter than ND controls (*p* = 0.02). This significance was not observed in females (*p* = 0.08). Furthermore, in men, circulating endotoxin and adiponectin were significantly higher in the T2DM group than ND controls (both *p*-values < 0.001). The opposite was observed in women, where endotoxin and adiponectin were significantly higher in ND controls than the T2DM group (*p*-values 0.002 and <0.001, respectively). Also, in women only, both HOMA-IR and TNF-α were significantly higher in the T2DM group than ND controls (*p*-values 0.03 and 0.02, respectively). The same parameters (HOMA-IR and TNF-α) were not significantly different in men T2DM and their ND case controls (Table 3).

### 3.3. Associations between Telomere Length and Endotoxin

Significant bivariate associations were noted for telomere length with studied parameters, for all participants, which included inverse associations with total and HDL cholesterol, CRP, and endotoxin. In ND control participants, TL was inversely associated with total cholesterol and endotoxin. TL was also positively associated with PAI-1 and TNF-α. Among T2DM participants, positive associations were observed between TL and glucose, HOMA-IR, adiponectin, resistin, and PAI-1, while significant inverse associations were elicited between LDL/HDL ratio, endotoxin, and CRP (Table 4).

Analysis of significant associations according to sex indicated a dimorphism, with a strong positive association between TL and age, as well as PAI-1 in men ND controls. Among T2DM males, TL was positively associated with glucose, triglycerides, HOMA-IR, resistin, and PAI-1, with an inverse significant association between TL and endotoxin, as well as CRP. Women ND controls however showed a significant inverse association between TL and WHR, as well as endotoxin, with positive association between TL and PAI-1. Only adiponectin showed a positive association with TL among the T2DM women (Table 4).

### 3.4. Significant Predictors of TL

Using all measured variables as independent variables, analysis determined the predictors for TL and endotoxin through stepwise linear regression. In all participants, endotoxin and triglycerides predicted 18% of the variance perceived in TL (*p* < 0.001). When stratified according to T2DM status, endotoxin and glucose were observed to be significant predictors of TL, while glucose and BMI were significant predictors of TL among ND controls. Stratification according to sex revealed that endotoxin significantly predicts 19% of the variances perceived in TL among male T2DM participants (*p* = 0.007). In male ND controls however, the significant predictors of TL were BMI and glucose (*p* = 0.001). Lastly in women, HbA1c was the significant predictor of TL while glucose was an important predictor of TL in female ND controls (*p*-values 0.01) (Table 5).

### 3.5. Effects of Medications on TL and Endotoxin

**Figure 1:**
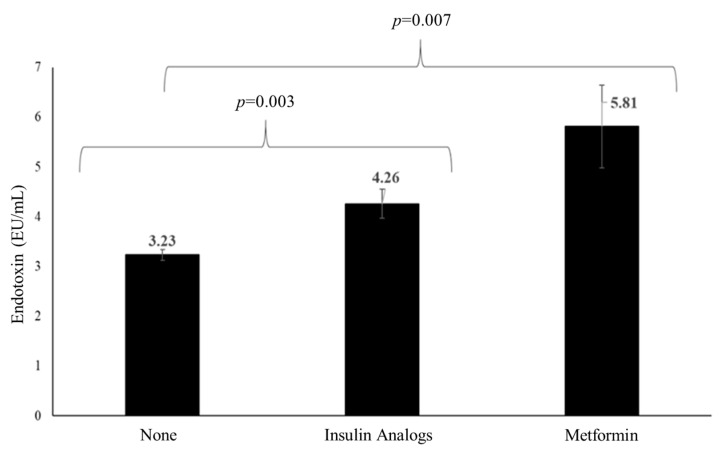
Differences in endotoxin levels according to anti-T2DM medications.

A univariate analysis of variance was performed to determine differences in the circulating levels of TL and endotoxin according to the medications provided to the participants, with age, BMI, and sex used as covariates. In this analysis, no difference in TL among statin versus non-statin users was noted, nor in aspirin versus non-aspirin users, anti-hypertensive versus non-anti-hypertensive users, as well as anti-T2DM versus non-anti-T2DM medication users. The same was apparent for endotoxin, with the exception of the use of anti-T2DM medication where endotoxin was lowest among non-users of any anti-T2DM medication (diet alone) (N = 696) compared with users of insulin analogs (N = 85) and metformin, respectively (N = 23) (Figure 1).

## 4. Discussion

It has been established that chronic diseases such as obesity and T2DM contribute to premature aging, and this study has added to our understanding of what may mediate the inflammatory insult in part to cause this effect, as this study shows for the first time that endotoxin appears to negatively influence TL in an adult Arabian population across varying degrees of insulin resistance. Furthermore, we have also shown differences in associations of TL among men and women with and without T2DM. The data from this study also highlighted that as endotoxin levels rise TL was observed to shorten, which, following stratification according to T2DM status and sex, revealed that these inverse associations were predominately observed in men with T2DM and ND control women, suggesting sexual dimorphism in premature biological aging as an independent factor from chronic sub-inflammation. 

Several previous studies to date have provided evidence for a relationship between TL and T2DM, a disease with a known higher rate of premature mortality [13,34,35], as well as it being noted that TL itself can provide prognostic information on the mortality risk of subjects with T2DM, which is also influenced by ethnicity [9,10,11,12,13,36]. Our data obtained here from another distinct ethnic group, Arab adults, extend the current literature by affirming the association between TL shortening and T2DM. This finding was extended by the association between gut-derived endotoxin and TL across both populations with and without T2DM and obesity. Furthermore, sex-specific analysis indicated that men with T2DM experienced advanced TL shortening associated with increased endotoxin. Despite the clear risk factors across the various groups, such as the degree of insulin resistance, obesity, hypertension, and metabolic state, male sex was noted and has been proposed as an independent predictor for increased telomere attrition. Previous studies have considered several hypotheses to explain this association [37,38]. Beyond the fact that for a given age and BMI men are noted to be at a higher metabolic risk than women prior to the menopause, it has been suggested that estrogen specifically plays a role in preventing shortening of TL by activating telomerase, due to the estrogen-responsive element that is present in telomerase reverse transcriptase (hTERT). Additionally, the antioxidant properties of estrogen protect telomeres from oxidative stress [39], which leads to lower reactive oxygen species (ROS), a proponent of accelerated telomere attrition [40,41]. Beyond estrogen, other factors that contribute to metabolic dysfunction can influence TL attrition, as noted in this study, which include CRP, lipids, and endotoxin. Previous studies examining CRP, as a sensitive marker of inflammation, have shown a significant correlation with TL [42,43]. Our study identified a strong inverse correlation between elevated CRP and TL in T2DM men, which further highlighted the continued presence of chronic low-grade inflammation in association with TL reduction. Beyond CRP as a marker of inflammation, studies have indicated that CRP can directly mediate the inactivation of telomerase and as such has been considered to promote early senescence [44], which may also explain the significant association between CRP and reduced TL in our T2DM male participants. The presence of sub-clinical systemic inflammation in the T2DM subjects of our study was also confirmed by increased PAI-1 levels as well as by resistin, which was significantly raised in T2DM men. Resistin has been regarded as an important link between obesity and T2DM from our own previous studies, suggesting how central obesity and abdominal adipocytes play a role in promoting resistin and other pro-inflammatory cytokines via the nuclear factor–κB signaling pathway [45,46], which has also been noted in human macrophages and peripheral mononuclear cells [47]. These pro-inflammatory systemic adipokines can also mediate pancreatic β-cell dysfunction, which can lead to a reduction in TL in pancreatic β-cells to impair insulin secretion [48], which is more pronounced in disturbed metabolic states.

In contrast, the anti-inflammatory adipokine adiponectin was noted to show a positive association with TL in T2DM participants, indicating that adiponectin could represent a positive influence to slow down aging as has been noted in a Japanese centenarian cohort study examining TL and adiponectin [49]. However, despite the apparent benefit of adiponectin in T2DM patients, in the long term this may be negated due to the overriding pro-inflammatory milieu. In further analysis of the adiponectin data, it was also observed that women with T2DM had lower adiponectin levels than their ND counterparts, although T2DM women were noted to show adiponectin positively associated with TL and the levels were higher in women than men in the present study, which affirms previously noted sex- and ethnicity-specific differences in other studies [50,51]. Previous studies have reported hypoadiponectinemia to act as a biomarker for insulin resistance and an independent risk factor for cardiovascular diseases and T2DM [52]. Other determinants that have been linked to an increased rate of TL shortening as well as increased cardiovascular risk include obesity, hypertension, deranged fasting plasma glucose, and high triglyceride with or without low HDL levels [53]. Our data align with a recent study undertaken in a multiethnic Asian population, which reported ethnic variation in the association between obesity indices (BMI and visceral fat area) and short TL in T2DM patients [54]. In Saudi Arabia, the overall prevalence of overweight, obesity, and severe obesity in all age groups was identified to be 23.1%, 9.3%, and 2%, respectively [55]. The BMI of T2DM subjects of the present study was comparable to that of the ND control group and indicated an overall prevalence of obesity, which did not differ between the groups. However, significant dyslipidemia was observed in our T2DM group, which has been previously noted in the literature [13]. An important observation from our study was the strong inverse association of total and LDL cholesterol with TL. It is known that elevated cholesterol levels can cause excessive generation of ROS, which increases cell injury and proliferation, resulting in premature cellular aging [56]. Thus, our data provide evidence for the rationale of hypercholesterolemia giving rise to premature aging in conjunction with other inflammatory factors.

A notable observation in this study was the strong inverse association between reduced TL and plasma endotoxin concentration in T2DM men but not in T2DM women. Meanwhile, as anticipated, endotoxin was higher in T2DM men than in their ND counterparts. This sex-specific finding for endotoxin has been observed by our team in other ethnic groups, including Arabian subjects [48,57], but the association with TL is novel. Whilst men are at a greater metabolic risk for a given age and BMI, the sexual dimorphism in their innate immune response may have a more profound impact on TL in men [58]. Another putative explanation for the difference could be the type of immune response, which is different between men and women; generally, men are more predisposed to infection and sepsis, whereas women are more likely to develop autoimmune diseases [59].

Our study has some limitations. Firstly, the study was observational, and causal inference cannot be determined. Longitudinal analysis of TL and endotoxin could strengthen the present findings. Secondly, the long-term influence of T2DM medications could not be addressed, which appears to influence circulating endotoxin and TL shortening in the T2DM group. Despite these caveats, the findings of the study are robust given its sample size and provide an avenue for further investigation as to whether manipulation of gut endotoxin can reverse biological aging.

## 5. Conclusions

In summary, these current studies continue to affirm that chronic sub-inflammation exacerbates premature biological aging, highlighting that gut-derived circulating endotoxin and triglycerides predict 18% of the variance in TL. Moreover, stratification of the participants according to metabolic state and sex also shows that endotoxin may further predict 19% of the variance denoted in TL among male T2DM participants. In conclusion, endotoxin can be seen as another important influence on premature aging as well as metabolic state, lipids, CRP, and gender among Arab adults. Future studies that are able to utilize interventions to manipulate the gut microbiome in metabolic diseases may be useful to mitigate the impact on telomere length and premature aging.

## Figures and Tables

**Table 1:** Clinical characteristics of participants.

Parameters	All			Men			Women		
	ND Control	T2DM	*p*-Value	ND Control	T2DM	*p*-Value	ND Control	T2DM	*p*-Value
N	417	387		183	202		234	185	
Obese (%)	207 (54.5)	195 (52.4)	0.85	67 (39.4)	73 (36.1)	0.57	140 (66.7)	122 (67.8)	0.47
Hypertensive (%)	129 (30.9)	165 (42.6)	0.001	53 (29.0)	77 (38.1)	0.07	76 (32.5)	88 (47.6)	0.002
Insulin Analogs (%)	-	85 (10.6)		-	48 (12.5)		-	37 (8.8)	
Metformin (%)	-	23 (2.9)		-	14 (3.6)		-	9 (2.1)	
Statins (%)	-	17 (2.1)		-	13 (3.4)		-	4 (1.0)	
Antihypertensive (%)	11 (2.6)	31 (8.0)	<0.001	5 (2.7)	16 (7.9)	<0.001	6 (2.6)	15 (8.1)	<0.001
Aspirin (%)	4 (0.2)	32 (8.3)	<0.001	3 (1.5)	23 (12.6)	<0.001	1 (0.4)	9 (4.9)	<0.001

Note: Data presented as N (%). Significant at *p* < 0.05.

**Table 2:** Anthropometrics and lipid profile of participants.

Parameters	All			Men			Women		
	ND Control	T2DM	*p*-Value	ND Control	T2DM	*p*-Value	ND Control	T2DM	*p*-Value
N	417	387		183	202		234	185	
Age (years)	55.7 ± 7.4	58.7 ± 8.1	<0.001	55.9 ± 8.4	60.9 ± 8.4	<0.001	55.5 ± 6.5	56.3 ± 6.9	0.26
BMI (kg/m^2^)	30.9 ± 5.6	30.6 ± 5.4	0.52	28.5 ± 4.7	29.1 ± 4.8	0.27	32.8 ± 5.5	32.3 ± 5.5	0.36
WHR	0.94 ± 0.1	0.96 ± 0.1	<0.001	0.96 ± 0.07	1.0 ± 0.06	<0.001	0.91 ± 0.09	0.91 ± 0.07	0.96
SBP (mmHg)	125.1 ± 14.3	132.4 ± 14.5	<0.001	125.9 ± 13.9	132.3 ± 12.9	<0.001	124.5 ± 14.5	132.4 ± 15.9	<0.001
DBP (mmHg)	77.9 ± 9.6	80.2 ± 8.7	0.001	79.8 ± 7.8	81.2 ± 6.0	0.09	76.4 ± 10.5	79.3 ± 10.6	0.007
Total Cholesterol	5.04 ± 1.1	5.07 ± 1.2	0.64	5.1 ± 1.2	4.9 ± 1.1	0.28	4.9 ± 0.9	5.2 ± 1.2	0.06
HDL Cholesterol	1.0 ± 0.3	0.99 ± 0.3	0.88	0.84 ± 0.3	0.92 ± 0.3	0.004	1.1 ± 0.3	1.1 ± 0.2	0.18
LDL Cholesterol	3.2 ± 0.9	3.1 ± 0.9	0.46	3.4 ± 1.0	3.1 ± 0.9	0.02	3.1 ± 0.8	3.2 ± 0.9	0.22
LDL/HDL ratio	3.7 ± 2.5	3.5 ± 1.5	0.13	4.4 ± 2.1	3.7 ± 1.6	<0.001	3.2 ± 2.6	3.2 ± 1.3	0.86
Triglycerides #	1.5 (1.2–2.1)	1.7 (1.3–2.3)	0.002	1.7 (1.3–2.3)	1.7 (1.2–2.3)	0.61	1.4 (1.1–2.0)	1.7 (1.3–2.4)	<0.001

Note: Data presented as mean ± SD, median (1st–3rd) percentile, and mean (95%CI), median (25th–75th) percentile change for Gaussian and non-Gaussian variables; # denotes non-Gaussian variable, presented as median. ND, non-diabetic; BMI, body mass index; WHR, waist–hip ratio; SBP, systolic blood pressure; DBP, diastolic blood pressure; HDL, high-density lipoprotein; LDL, low-density lipoprotein. Significant at *p* < 0.01.

**Table 3:** Telomere length, glycemic profile, and pro-inflammatory markers in participants with or without T2DM.

Parameters	All			Men			Women		
	ND Control	T2DM	*p*-Value	ND Control	T2DM	*p*-Value	ND Control	T2DM	*p*-Value
N	417	387		234	185		183	202	
TL (BP)	5880 ± 1464	5554 ± 1346	<0.001	5830 ± 1705	5435 ± 1380	0.02	5912.3 ± 1290	5686 ± 199	0.08
Glucose (mmol/L)	5.7 ± 0.9	10.2 ± 3.1	<0.001	5.8 ± 0.9	10.0 ± 3.0	<0.001	5.6 ± 0.8	10.3 ± 3.2	<0.001
Insulin (μU/mL)	11.1 (6.9–16.3)	10.1 (6.5–16.1)	0.14	10.3 (6.8–16.8)	9.7 (6.3–16.0)	0.812	11.8 (7.2–16.3)	10.4 (6.5–16.1)	0.087
HOMA-IR	2.8 (1.7–4.5)	4.2 (2.7–7.6)	<0.001	2.5 (1.5–4.6)	3.9 (2.7–7.6)	<0.001	2.9 (1.9–4.4)	4.3 (2.7–7.4)	<0.001
Endotoxin (IU/mL)	3.0 (1.8–4.8)	2.5 (1.7–4.1)	0.03	2.6 (1.7–4.1)	2.5 (1.7–4.6)	0.56	3.2 (1.8–4.9)	2.4 (1.8–3.8)	0.03
Adiponectin (ug/mL)	13.5 (5.4–24.9)	13.9 (8.5–20.2)	0.38	3.4 (1.0–13.1)	11.8 (8–17)	<0.001	18.6 (11.9–34)	15.8 (10.1–25)	0.002
Resistin (ng/mL)	27.3 (2.7–114)	28.4 (20–41)	0.97	2.2 (1.2–10.1)	25.7 (18.2–38)	<0.001	96.9 (31.4–168)	30.9 (21.2–43)	<0.001
PAI-1 (ng/mL)	6.9 (0.3–10.4)	10.1 (7.4–12.7)	<0.001	0.3 (0.1–4.4)	10.1 (7.4–17)	<0.001	9.4 (6.8–12.4)	10.0 (7.6–12.7)	0.048
Leptin (ng/mL)	16.6 (6.4–32.2)	14.7 (8.3–26.4)	0.56	10.7 (4.7–23.8)	10.8 (6.2–16.7)	0.95	22.3 (10.5–37)	20.9 (12.4–33)	0.99
ANG-II (pg/mL)	0.9 (0.4–1.2)	0.2 (0.1–0.4)	<0.001	1.0 (0.6–1.3)	0.2 (0.1–0.5)	<0.001	0.20 (0.08–0.3)	0.21 (0.1–0.4)	0.18
TNF-α (pg/mL)	2.4 (1.3–6.1)	1.3 (0.9–2.1)	<0.001	3.7 (1.5–7.2)	1.3 (0.9–1.9)	<0.001	2.0 (1.3–3.4)	1.4 (0.9–2.1)	<0.001
CRP (ug/mL)	2.0 (0.8–4.5)	2.9 (0.9–6.0)	0.06	2.8 (0.9–6.5)	3.0 (1.5–6.0)	0.55	1.5 (0.7–2.5)	2.8 (0.2–5.6)	0.02

Note: Data presented as mean ± SD, median (1st–3rd) percentile, and mean (95%CI), median (25th–75th) percentile change for Gaussian and non-Gaussian variables. ND, non-diabetic; TL, telomere length; HOMA-IR, Homeostatic Model Assessment for Insulin Resistance; PAI-1, plasminogen activator inhibitor-1; ANG-II, angiotensin II; TNF-α, tumor necrosis factor α; CRP, C-reactive protein. *P*-values are obtained from independent sample *t*-test and Mann–Whitney U test for Gaussian and non-Gaussian variables, respectively. *p*-value < 0.05 considered significant.

**Table 4:** Significant associations of TL with measured parameters.

Parameters	All	All		Males		Females	
		ND Control	T2DM	ND Control	T2DM	ND Control	T2DM
N (M/F)	804	417 (183/234)	387 (202/185)	183	202	234	185
Age (years)				0.24 **			
WHR						−0.16 *	
Glucose (mmol/L)			0.20 **		0.24 **		
Total Cholesterol	−0.08 *	−0.10 *					
HDL Cholesterol	−0.08 *						
LDL/HDL ratio			−0.13 *				
Triglycerides #					0.15 *		
HOMA-IR			0.14 *		0.20 *		
Endotoxin (IU/mL)	−0.17 **	−0.27 **	−0.18 **		−0.24 **	−0.29 **	
Adiponectin (ug/mL)			0.18 *				0.23 *
Resistin (ng/mL)			0.15 *		0.20 *		
PAI-1 (ng/mL)		0.18 *	0.25 **	0.32**	0.27 **	0.25 *	
TNF-α (pg/mL)		−0.15 *					
CRP (ug/mL)	−0.14 *		−0.15 *		−0.25 **		

Note: Data presented as coefficient (R); # denotes not normal; * denotes significance at 0.05 level; ** denotes significance at 0.01 level. Parameters not included in the table denote no significant correlations in all groups.

**Table 5:** Significant predictors of TL and endotoxin.

Parameters	N	Significant Predictors of TL
All Participants	804	Endotoxin, Triglycerides, BMI R^2^ = 0.18; *p* < 0.001
T2DM	387	Endotoxin, Glucose R^2^ = 0.21; *p* = 0.002
ND Control	417	Glucose, BMI R^2^ = 0.56; *p* < 0.001
Male T2DM	185	Endotoxin R^2^ = 0.19; *p* = 0.007
Male ND Control	235	BMI and Glucose R^2^ = 0.76; *p* = 0.001
Female T2DM	202	HbA1c R^2^ = 0.68; *p* = 0.14
Female ND Control	183	Glucose R^2^ = 0.13; *p* = 0.01

Note: Data presented as adjusted R^2^. Independent variables: age, BMI, WHR, glucose, triglycerides, adiponectin, resistin, leptin, PAI-1, TNF-α, insulin, 25(OH)D, ANGII, CRP, TL, and endotoxin.

## Data Availability

The research data from the study may be available from the corresponding author on reasonable request.
